# Alexithymia in neurodevelopmental populations: a scoping review of Down syndrome, autism spectrum disorder, and dual diagnosis

**DOI:** 10.3389/fpsyg.2026.1705694

**Published:** 2026-06-05

**Authors:** Nasr Chalghaf, Imed Chokri, Wissem Dhahbi, Halil Ibrahim Ceylan, Valentina Stefanica, Noomen Guelmami, Ismail Dergaa

**Affiliations:** 1 Department of Education, High Institute of Sport, and Physical Education of Gafsa, University of Gafsa, Gafsa, Tunisia; 2 Department of Health Sciences (DiSSal), Postgraduate School of Public Health, University of Genoa, Genoa, Italy; 3 High Institute of Sport and Physical Education, University of Sfax, Sfax, Tunisia; 4 Research Laboratory, Education, Motricity, Sport and Health (EM2S), High Institute of Sport and Physical Education of Sfax, University of Sfax, Sfax, Tunisia; 5 Research Unit “Sport Sciences, Health and Movement”, Higher Institute of Sports and Physical Education of Kef, University of Jendouba, Kef, Tunisia; 6 Training Department, Police College, Police Academy, Doha, Qatar; 7 Faculty of Sports Sciences, Physical Education of Sports Teaching Department, Atatürk University, Erzurum, Turkey; 8 Faculty of Sciences, Physical Education and Informatics, Department of Physical Education and Sport, Pitesti University Center, National University of Science and Technology Politehnica Bucharest, Pitesti, Romania; 9 Department of Social Sciences, High Institute of Sports and Physical Education of Kef, University of Jendouba, El Kef, Tunisia

**Keywords:** affective disorders, behavioral symptoms, communication disorders, empathy, mental health services, neuropsychological tests, psychometrics, social cognition

## Abstract

**Background:**

Alexithymia, characterized by difficulties in identifying and describing emotions, affects ~10% of the general population. Prevalence is substantially higher in individuals with neurodevelopmental disorders.

**Objective:**

To systematically map existing research on alexithymia across Down syndrome, autism spectrum disorder, and dual diagnosis populations, synthesize current knowledge, and identify critical research gaps informing future investigation priorities.

**Methods:**

Following Arksey and O'Malley's framework and PRISMA-ScR guidelines, searches were conducted across MEDLINE, PsycINFO, EMBASE, CINAHL, and Cochrane databases from inception to December 2024. Studies examining alexithymia, emotional processing, or emotional awareness in DS, ASD, or DS-ASD populations underwent selection and narrative synthesis employing thematic analysis.

**Results:**

Database searches yielded 2,847 records, of which 55 studies met inclusion criteria spanning 1994-2024. Literature demonstrates research imbalances, with extensive evidence on ASD contrasted with minimal investigation on DS and the absence of research on dual diagnoses. Six major themes emerged: prevalence disparities with ASD populations showing 49.9% weighted mean prevalence vs. unestablished DS rates; assessment limitations with current tools lacking intellectual disability validation; intervention approaches demonstrating moderate effectiveness (*d* = 0.65) in limited populations; neurobiological correlates implicating emotion processing networks; developmental trajectory gaps across the lifespan; and clinical implications for therapeutic engagement and quality of life. Seven research gaps were identified, including the absence of validated assessment tools for populations with intellectual disabilities and the lack of longitudinal developmental studies.

**Conclusion:**

Current evidence reveals research imbalances with extensive ASD literature contrasting with the absence of DS research, limiting understanding and evidence-based practice. The lack of dual diagnosis investigation represents a gap affecting 16-18% of individuals with DS. Priorities include developing assessment methodologies for intellectual disability populations, establishing DS alexithymia prevalence, and investigating population-specific intervention approaches to optimize therapeutic outcomes and quality of life across the neurodevelopmental spectrum.

**Systematic review registration:**

https://doi.org/10.17605/OSF.IO/K8UEC, identifier: OSF.IO/K8UEC.

## 1 Introduction

Alexithymia affects an estimated 33-63% of individuals with autism spectrum disorder (Kinnaird et al., 2019b; Poquérusse et al., 2018; Vaiouli et al., 2022). It contributes to therapeutic resistance (Spain et al., 2015; Conner et al., 2019), social isolation (Morie et al., 2019; Vuillier et al., 2020), and reduced quality of life across neurodevelopmental populations (Costa et al., 2020; Moseley et al., 2019). However, alexithymia remains virtually unstudied in Down syndrome despite affecting an estimated 250,000 individuals worldwide (Sherman et al., 2007; De Graaf et al., 2015), representing a critical gap with immediate implications for clinical practice, intervention development, and evidence-based care delivery (Lai et al., 2013; Munoz-Solomando et al., 2008).

The understanding of alexithymia has evolved substantially since Sifneos' 1970s conceptualization (Sifneos, 1973), establishing this multidimensional construct as a transdiagnostic risk factor characterized by difficulty identifying feelings (DIF), difficulty describing feelings (DDF), and externally oriented thinking (EOT), which directs attention away from internal emotional states (Taylor et al., 1999, 2016). Clinical significance encompasses therapeutic engagement (Luminet et al., 2021; Grynberg et al., 2012; Cameron et al., 2014; Gratz and Tull, 2010), interpersonal relationships (Mattila et al., 2006; Honkalampi et al., 2000), and adaptive functioning across diverse populations (Westwood et al., 2017; Karukivi and Saarijärvi, 2014). Meta-analytic evidence indicates a prevalence of ~10% in general populations (Bagby et al., 1994a,b), with substantially higher prevalence in psychiatric and medical conditions (Marchesi et al., 2014; Tulipani et al., 2010), particularly neurodevelopmental disorders, where rates may exceed 50% (Kinnaird et al., 2019b; Poquérusse et al., 2018).

Research in neurodevelopmental populations reveals considerable complexity (Berthoz and Hill, 2005; Gaigg et al., 2018), with evidence suggesting that emotional processing difficulties previously attributed to core disorder characteristics may instead reflect co-occurring alexithymic features, which require distinct therapeutic approaches (Cook et al., 2013; Bird and Cook, 2013). This paradigm shift proves transformative in autism research (Hill et al., 2004; Silani et al., 2008), where the “alexithymia hypothesis” proposes that socio-emotional difficulties traditionally considered intrinsic to autism stem from alexithymic traits (Brewer et al., 2015; Bird et al., 2010), challenging assumptions about emotional processing in neurodevelopmental conditions (Shah et al., 2016; Heaton et al., 2012). Systematic reviews and meta-analyses consistently demonstrate that ~50% of autistic individuals exhibit clinically significant alexithymia levels (Kinnaird et al., 2019b; Poquérusse et al., 2018; Milosavljevic et al., 2016), with effect sizes indicating large magnitude differences compared to neurotypical populations (Griffin et al., 2016; Samson et al., 2012). However, this research landscape reveals methodological heterogeneity (Lombardo et al., 2007; Albantakis et al., 2020), assessment limitations (Williams and Gotham, 2021; Kinnaird et al., 2019a), and disparities in population focus that complicate a comprehensive understanding and clinical application (Jenner et al., 2023; Shaffer et al., 2023). In particular, the Toronto Alexithymia Scale-20 (TAS-20) (Bagby et al., 1994a) demonstrates questionable validity when applied to individuals with intellectual disabilities or autism (Greene et al., 2020; Kooiman et al., 2002). This raises concerns about measurement accuracy and clinical utility (Levant et al., 2009; Rosenberg et al., 2016).

Seven critical research gaps impede a comprehensive understanding and clinical application (Bagby et al., 2020; Montebarocci and Surcinelli, 2018). First, a striking research imbalance exists between autism and Down syndrome populations (Uljarevic and Hamilton, 2013; Losh and Capps, 2006), with extensive ASD literature contrasting sharply with minimal DS-specific investigation despite Down syndrome representing the most common genetic cause of intellectual disability affecting ~1 in 700–1,000 births globally (Sherman et al., 2007; De Graaf et al., 2015). Second, methodological heterogeneity limits synthesis (Brewer et al., 2016; Gormley et al., 2022) due to inconsistent operational definitions, varied assessment approaches, and diverse outcome measures, thereby preventing comparative analysis (Buckley et al., 2020; Maulik et al., 2011). Third, the virtual absence of research examining alexithymia in dual diagnosis populations (DS-ASD) represents a profound knowledge gap (Channell et al., 2015; Moss et al., 2013), given that 16-18% of individuals with Down syndrome also meet criteria for autism spectrum disorder according to recent meta-analytic evidence (DiGuiseppi et al., 2010; Capone et al., 2005). Fourth, developmental considerations remain unexplored (Frye et al., 2015; Billeci et al., 2016), with research focusing on adults while neglecting the emergence of alexithymic features across the lifespan in neurodevelopmental populations (Mazefsky et al., 2013; Rieffe et al., 2014). Fifth, intervention research specifically targeting alexithymia in neurodevelopmental disorders remains extremely limited (Tsubaki and Shimizu, 2024; Gratz et al., 2014), despite growing evidence that alexithymic features may be modifiable through targeted therapeutic approaches (Neacsiu et al., 2010; Spain and Happé, 2020). Sixth, the implications of alexithymia for adaptive functioning, quality of life, and long-term outcomes require systematic investigation (Costa et al., 2019; Giannotti et al., 2020). Seventh, the validation and adaptation of assessment tools for populations with intellectual disability remain inadequate (Haviland and Reise, 1996; Haviland et al., 2002), which limits research progress and clinical application (Montebarocci et al., 2004; Kench and Irwin, 2000).

To address these fundamental knowledge gaps, this scoping review aimed to systematically map existing research on alexithymia across Down syndrome, autism spectrum disorder, and dual diagnosis populations. Our specific objectives were to: (1) comprehensively identify and characterize the current literature landscape examining alexithymia in these populations; (2) synthesize existing knowledge through rigorous thematic analysis to understand key findings, prevalence patterns, and methodological approaches; (3) identify and analyze critical research gaps to inform future investigation priorities and funding allocation; (4) examine assessment approaches and their appropriateness for neurodevelopmental populations with particular attention to psychometric properties and clinical utility; (5) explore intervention strategies and their evidence base to identify practical approaches and development needs; and (6) provide evidence-based recommendations for researchers, clinicians, and policymakers to advance understanding and optimize support for individuals with neurodevelopmental disorders and co-occurring alexithymia.

## 2 Methods

### 2.1 Study design and methodological framework

We carried out a scoping review following (Arksey and O'Malley 2005) and subsequent methodological developments (Levac et al., 2010; Pham et al., 2014). The review adhered to PRISMA-ScR guidelines (Tricco et al., 2018), ensuring transparent reporting. This approach enhances methodological rigor (Moher et al., 2009; Shamseer et al., 2015). The scoping review approach was selected to map the existing literature across diverse populations, examine the breadth of research activity, and identify knowledge gaps. This method emphasizes evidence mapping rather than evaluating intervention effectiveness through meta-analytic synthesis (O'Brien et al., 2016; Cooper C. et al., 2018).

This study was registered with the Open Science Framework (OSF), enhancing transparency and supporting reproducibility. The registration provides information on methods and objectives, supporting scientific collaboration (https://doi.org/10.17605/OSF.IO/K8UEC).

### 2.2 Research questions and scope definition

The primary research question guiding this scoping review was formulated according to established frameworks (O'Brien et al., 2016): “What is the current state of knowledge regarding alexithymia in individuals with Down syndrome, autism spectrum disorder, and dual diagnosis presentations?” Five secondary questions provided analytical focus: (1) What assessment approaches have been employed to measure alexithymia in these populations, and what are their psychometric properties and clinical utility? (2) What interventions have been developed and empirically tested for alexithymia in neurodevelopmental disorders, and what is their evidence base? (3) What are the key research gaps and methodological limitations constraining current literature? (4) How do prevalence patterns and characteristics of alexithymia vary across different neurodevelopmental populations? (5) What are the clinical implications and service delivery considerations for individuals with neurodevelopmental disorders and co-occurring alexithymia? The scope encompassed studies examining alexithymia, emotional processing difficulties, emotional awareness deficits, or related constructs in individuals with Down syndrome (DS), autism spectrum disorder (ASD), or dual diagnosis (DS-ASD) presentations (Cooper C. et al., 2018; Bramer et al., 2017).

### 2.3 Comprehensive search strategy

Systematic searches were conducted across five major electronic databases: MEDLINE (via PubMed), PsycINFO, EMBASE, CINAHL, and Cochrane Library from database inception to December 2024 (Rethlefsen et al., 2021; Godin et al., 2015). The search strategy was developed through consultation with research librarians and pilot testing to optimize sensitivity while maintaining specificity (Sampson et al., 2009; McGowan et al., 2016). Search terms were organized into three primary concept groups using Boolean operators: (1) alexithymia and emotional processing terms: “alexithymia,” “alexithymic,” “emotional processing,” “emotional awareness,” “emotional recognition,” “emotion regulation,” “difficulty identifying feelings,” “difficulty describing feelings,” “externally oriented thinking”; (2) neurodevelopmental disorder terms: “Down syndrome,” “trisomy 21,” “autism spectrum disorder,” “autism,” “Asperger syndrome,” “pervasive developmental disorder,” “intellectual disability,” “developmental disability”; and (3) study design terms: “prevalence,” “assessment,” “intervention,” “treatment,” “therapy,” “measurement,” “evaluation.” Truncation symbols (^*^) and wildcard characters (?) were employed to capture variant spellings and word forms (Bramer et al., 2016). Gray literature searches included conference proceedings, dissertation databases, and government reports to minimize publication bias (Paez, 2017; Schöpfel, 2010).

### 2.4 Study selection process and criteria

Study selection followed a two-stage screening process conducted by two independent reviewers with neurodevelopmental research experience (Shea et al., 2017; Higgins et al., 2011). Initial screening involved reviewing titles and abstracts against predefined criteria, followed by a full-text review of potentially relevant studies (Li et al., 2022; Veritas Health Innovation, 2022). Inclusion criteria were: (1) empirical studies examining alexithymia or related emotional processing constructs using validated assessment tools; (2) participants with Down syndrome, autism spectrum disorder, or dual diagnosis presentations; (3) any study design including cross-sectional, longitudinal, intervention, and qualitative investigations; (4) peer-reviewed publications in academic journals; (5) participants across all age groups from childhood through adulthood; (6) English language publications with accessible full text. Exclusion criteria included: (1) studies not examining target neurodevelopmental populations; (2) conference abstracts without corresponding full-text publications; (3) case reports or case series with fewer than five participants; (4) purely theoretical papers without empirical data; (5) studies examining emotional processing without specific alexithymia assessment; (6) duplicate publications or overlapping datasets. Disagreements were resolved through discussion and consultation with a third reviewer when consensus could not be reached (McHugh, 2012; Landis and The, 1977).

### 2.5 Data extraction and quality assessment

**Figure 1:**
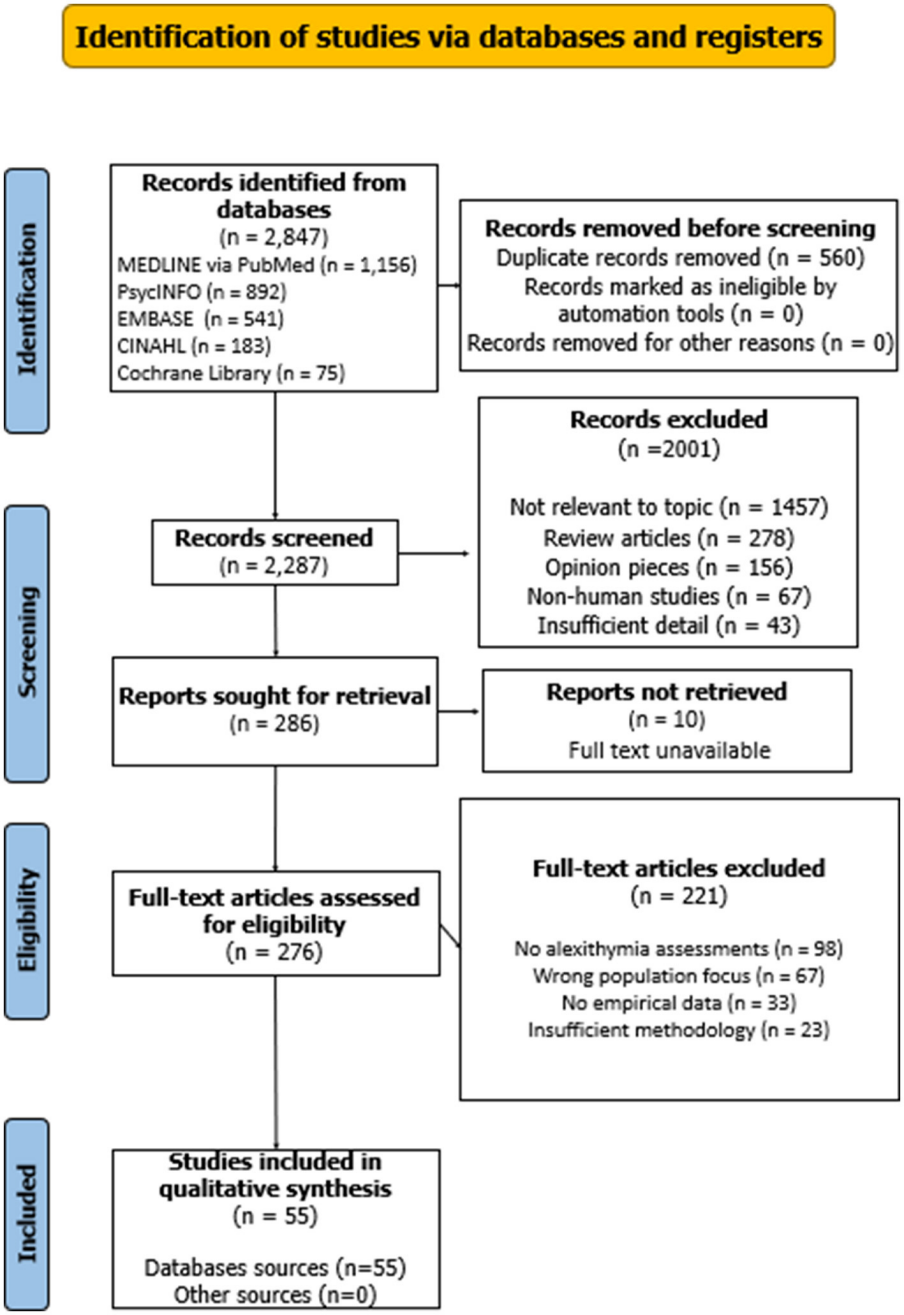
PRISMA-ScR flow diagram illustrating the identification, screening, eligibility, and inclusion of studies in the scoping review.

Data extraction employed a standardized form developed for this review and pilot-tested on included study subsets to ensure comprehensiveness and reliability (Liberati et al., 2009; Stroup et al., 2000). Extracted data elements included: (1) study characteristics: author, publication year, country, study design, sample size, funding source; (2) participant characteristics: age range, sex distribution, diagnostic criteria, intellectual functioning level, comorbid conditions; (3) alexithymia assessment: measures used, psychometric properties, administration method, outcome definitions; (4) key findings: prevalence rates, correlation coefficients, effect sizes, statistical significance levels; (5) methodological quality indicators: sampling methods, control group inclusion, confounding variable control, statistical analysis appropriateness. Quality assessment used a modified Newcastle-Ottawa Scale, adapted for diverse study designs (Wells et al., 2011; Modesti et al., 2016), which evaluated selection methods, group comparability, and the quality of outcome assessment (Figure 1).

### 2.6 Data synthesis and analytical approach

Data synthesis employed narrative analysis (Popay et al., 2006; Thomas and Harden, 2008), focusing on literature mapping rather than statistical aggregation. Thematic analysis identified patterns across studies (Braun and Clarke, 2006; Dixon-Woods et al., 2006), with themes emerging inductively while being anchored to the research questions. The analytical framework examined: (1) prevalence patterns across populations with attention to methodological factors influencing estimates; (2) assessment approaches including psychometric properties and appropriateness for target populations; (3) intervention strategies with analysis of effectiveness and population-specific adaptations; (4) neurobiological mechanisms and underlying pathways; (5) developmental trajectories and lifespan considerations; (6) clinical implications and service delivery considerations. Population-specific analyses were conducted for autism spectrum disorder, Down syndrome, and dual diagnosis groups, with cross-population comparisons highlighting similarities and differences in research approaches and findings.

## 3 Results

### 3.1 Search results and study selection

The search strategy identified 2,847 unique records across five electronic databases, with MEDLINE and PsycINFO contributing the largest proportions, followed by EMBASE, CINAHL, and the Cochrane Library. Following title and abstract screening, 286 studies proceeded to full-text review for eligibility assessment. After applying the inclusion and exclusion criteria, 55 studies met the inclusion criteria for the final synthesis. The study selection process is illustrated in the PRISMA-ScR flow diagram (Figure 1). Frequent exclusion reasons during full-text review were: absence of validated alexithymia or emotional processing measures; focus on other neurodevelopmental conditions outside the scope; insufficient empirical data for synthesis; and non-English-language publications without available translations.

### 3.2 Study characteristics and literature landscape

The 55 included studies spanned publication years from 1994 to 2024, with accelerated research activity over the past decade. The geographic distribution revealed a concentration in high-income, English-speaking countries, with the United States, United Kingdom, Australia, and Canada contributing the majority of studies. Study designs primarily employed quantitative approaches, with qualitative and mixed methods comprising smaller proportions. Sample sizes varied widely, from small exploratory studies to extensive population-based investigations, reflecting diverse methodological approaches.

The literature revealed population-specific research imbalances that limit a comprehensive understanding. Autism spectrum disorder populations dominated the evidence base with extensive research documented in multiple systematic reviews (Kinnaird et al., 2019b; Poquérusse et al., 2018; Vaiouli et al., 2022). In contrast, Down syndrome populations showed remarkably limited research, with only minimal studies examining emotional processing in intellectual disability contexts. Dual diagnosis populations (DS-ASD) were completely absent from the literature. This research disparity represents a critical gap, given that Down syndrome affects ~250,000 individuals globally and represents the most common genetic cause of intellectual disability (Sherman et al., 2007; De Graaf et al., 2015).

### 3.3 Thematic synthesis results

#### 3.3.1 Theme 1: prevalence patterns and population disparities

The literature revealed disparities in research attention and prevalence documentation across target populations, with robust evidence in autism contrasting with a virtual absence of Down syndrome research (Table 1). Autism spectrum disorder populations demonstrated the most comprehensive evidence base, supported by multiple systematic reviews and meta-analyses providing convergent evidence of substantially elevated alexithymia prevalence (Kinnaird et al., 2019b; Poquérusse et al., 2018; Milosavljevic et al., 2016). The landmark systematic review by Kinnaird and colleagues synthesized data from 15 studies, revealing a weighted mean alexithymia prevalence of 49.9% (95% CI: 46.4–53.4%) in ASD populations compared to 4.9% (95% CI: 3.2–6.6%) in neurotypical controls, representing a 10.2-fold increased prevalence rate (Kinnaird et al., 2019b). Individual studies within ASD populations have consistently reported prevalence ranges from 33.3% to 63.0%, with methodological factors, including assessment tools, diagnostic criteria, sample characteristics, and age ranges, contributing to the observed variation (Griffin et al., 2016; Samson et al., 2012; Lombardo et al., 2007).

**Table 1:** Alexithymia prevalence across neurodevelopmental populations.

Population	Research volume	Assessment tools	Prevalence estimates	Evidence quality	Methodological limitations
Autism spectrum disorder	Extensive literature base (55 studies; Kinnaird et al., 2019b; Poquérusse et al., 2018; Vaiouli et al., 2022)	TAS-20, PAQ, TSIA, observer measures (Bagby et al., 1994a; Preece et al., 2018; Bagby et al., 2006)	49.9% weighted mean (95% CI: 46.4-53.4%) (Kinnaird et al., 2019b); Range: 33.3-63.0% across included datasets	Multiple systematic reviews and meta-analyses (Kinnaird et al., 2019b; Poquérusse et al., 2018; Vaiouli et al., 2022)	Discriminant validity concerns with TAS-20 (Preece et al., 2024; Leising et al., 2009); Potential distress confounding (Maulik et al., 2011; Channell et al., 2015)
Down syndrome	Minimal research activity	Modified instruments, observer-rated scales (Haviland and Reise, 1996; Haviland et al., 2002)	Prevalence undetermined; insufficient data for estimation	Inadequate evidence base	Absence of validated assessment tools (Haviland and Reise, 1996; Haviland et al., 2002); Cognitive/communication barriers (Levant et al., 2006; Sinason, 2005)
Dual diagnosis (DS-ASD)	Complete research absence	No validated approaches	Prevalence unknown; No empirical data	No available evidence	The population comprises 16–18% of DS individuals (DiGuiseppi et al., 2010; Capone et al., 2005), yet remains unexamined
General population	Well-established research foundation (Bagby et al., 1994a,b)	Primarily TAS-20 (Bagby et al., 1994a,b); Standardized protocols	~10% (Bagby et al., 1994a,b); Consistent cross-cultural findings	Large-scale population studies (Bagby et al., 1994a,b)	Standard measures demonstrate appropriate psychometric properties

ASD, autism spectrum disorder; DS, Down syndrome; DS-ASD, dual diagnosis (Down Syndrome with co-occurring Autism Spectrum Disorder); CI, confidence interval; TAS-20, Toronto alexithymia scale-20; PAQ, Perth Alexithymia questionnaire; TSIA, Toronto structured interview for Alexithymia.

Evidence quality was assessed using modified Newcastle-Ottawa Scale criteria adapted for diverse study designs, incorporating selection bias, comparability, and outcome assessment domains.

Range variations reflect methodological heterogeneity, including diagnostic criteria (DSM-IV vs. DSM-5), assessment tools employed, participant age ranges, intellectual functioning levels, and cultural contexts across studies.

In contrast, Down syndrome populations demonstrated research paucity, with our search revealing extremely limited investigation of alexithymia or emotional processing difficulties specifically in individuals with Down syndrome. This represents a critical knowledge gap given that Down syndrome affects ~1 in 700–1000 births globally and constitutes the most common genetic cause of intellectual disability (Sherman et al., 2007; De Graaf et al., 2015). The few available studies that examined emotional processing in intellectual disability contexts used modified assessment approaches to address cognitive and communication considerations but provided insufficient evidence for reliable prevalence estimates or meaningful comparisons with other neurodevelopmental populations.

Dual diagnosis populations (DS-ASD) represented the most understudied group despite comprising 16-18% of individuals with Down syndrome according to meta-analytic evidence (DiGuiseppi et al., 2010; Capone et al., 2005). No studies were identified that specifically examined alexithymia in DS-ASD populations, despite this group potentially experiencing compounded emotional processing challenges requiring specialized assessment and intervention approaches.

#### 3.3.2 Theme 2: assessment approaches and methodological limitations

Assessment of alexithymia across neurodevelopmental populations has revealed methodological challenges and validity limitations that compromise cross-study synthesis and clinical application (Table 2) (Haviland et al., 2000; Way et al., 2007). The Toronto Alexithymia Scale-20 (TAS-20) emerged as the predominant measure across ASD studies (Bagby et al., 1994a), despite growing evidence of questionable appropriateness for neurodevelopmental populations and significant psychometric limitations (Greene et al., 2020; Kooiman et al., 2002).

**Table 2:** Assessment tools for alexithymia in neurodevelopmental populations.

Assessment instrument	Target populations	Age requirements	Psychometric properties	Critical limitations	Available modifications
Toronto Alexithymia Scale-20 (TAS-20) (Bagby et al., 1994a)	ASD populations, general samples	≥16 years (Bagby et al., 1994a)	Cronbach's α = 0.81–0.86; Good test-retest reliability (Bagby et al., 1994a,b); Three-factor structure	Discriminant validity issues with DIF subscale (Preece et al., 2024; Leising et al., 2009); Inappropriate for intellectual disability (Haviland and Reise, 1996; Haviland et al., 2002); Distress contamination (Gignac et al., 2005; Loas et al., 2017)	Simplified language versions (Rieffe et al., 2008); Visual adaptations under development
Perth Alexithymia Questionnaire (PAQ) (Preece et al., 2018)	Limited ASD research	≥18 years (Preece et al., 2018)	Cronbach's α = 0.95; Superior discriminant validity (Preece et al., 2018, 2020); Clean factor structure	Minimal validation in neurodevelopmental populations (Becerra et al., 2021); Limited research utilization	Picture-based versions in development (Rieffe et al., 2008); Caregiver-report adaptations proposed
Toronto Structured Interview for Alexithymia (TSIA) (Bagby et al., 2006)	ASD, some intellectual disability populations	≥16 years (Bagby et al., 2006)	Good inter-rater reliability (Bagby et al., 2006); Semi-structured format	Extensive training requirements (Bagby et al., 2006); Time-intensive administration; Limited scalability	Visual support materials available (Rieffe et al., 2008); Modified protocols for cognitive impairment
Observer-rated measures (Haviland and Reise, 1996; Haviland et al., 2002)	Intellectual disability populations	All age ranges (Haviland and Reise, 1996)	Variable psychometric properties (Haviland et al., 2002; Montebarocci et al., 2004); Context-dependent reliability	Observer bias potential (Haviland et al., 2002); Limited validation studies; Standardization gaps	Caregiver report versions (Montebarocci et al., 2004); Behavioral observation protocols
Pediatric measures (Way et al., 2007; Rieffe et al., 2008)	Limited ASD validation studies	8–18 years (Way et al., 2007)	Preliminary validation evidence (Way et al., 2007; Rieffe et al., 2008); Age-appropriate content	Age-restricted utility (Way et al., 2007); Reading ability requirements; Limited normative data	Picture-based adaptations (Rieffe et al., 2008); Technology-enhanced formats proposed

DIF, difficulty identifying feelings; α, Cronbach's alpha coefficient; ID, intellectual disability.

The psychometric properties reported represent ranges across validation studies in neurotypical populations, unless otherwise specified. Reliability coefficients may not generalize to neurodevelopmental populations due to concerns about measurement invariance.

Critical limitations encompass both psychometric deficiencies (validity, reliability, factor structure instability) and practical implementation barriers (cognitive demands, communication requirements, cultural appropriateness).

Multiple investigations highlighted discriminant validity problems with the TAS-20, particularly the Difficulty Identifying Feelings (DIF) subscale, which showed cross-loadings with general psychological distress measures rather than distinct alexithymic features (Preece et al., 2024; Leising et al., 2009; Gignac et al., 2005). This validity concerns proved especially pronounced in autism populations, where elevated distress levels may artificially inflate alexithymia scores, leading to overestimation of prevalence and misattribution of emotional difficulties (Loas et al., 2017).

The Perth Alexithymia Questionnaire (PAQ) demonstrated superior discriminant validity compared to the TAS-20, with all subscales loading cleanly on alexithymia factors without cross-contamination from distress factors (Preece et al., 2018, 2020). However, PAQ validation in neurodevelopmental populations remains extremely limited, with only eight studies employing this measure in ASD populations and no validation studies in intellectual disability or Down syndrome populations (Becerra et al., 2021).

A gap emerged regarding assessment tools suitable for individuals with intellectual disabilities (Haviland and Reise, 1996; Haviland et al., 2002). Current self-report measures universally require cognitive and language capabilities that may exceed the abilities of many individuals with Down syndrome or severe autism spectrum disorder (Levant et al., 2006; Sinason, 2005). Alternative approaches, including observer-rated scales and structured interviews, show promise but require extensive validation and standardization before clinical implementation (Montebarocci et al., 2004; Kench and Irwin, 2000).

#### 3.3.3 Theme 3: intervention approaches and treatment effectiveness

Intervention research targeting alexithymia in neurodevelopmental populations has demonstrated emerging evidence of treatment effectiveness, with gaps in population-specific approaches and limited long-term follow-up data (Table 3) (Tsubaki and Shimizu, 2024; Gratz et al., 2014). The majority of intervention studies focused on general alexithymic populations rather than neurodevelopmental-specific samples, significantly limiting direct applicability to ASD, DS, or dual diagnosis individuals (Neacsiu et al., 2010; Spain and Happé, 2020).

**Table 3:** Intervention approaches for alexithymia in neurodevelopmental populations.

Intervention modality	Evidence base	Target demographics	Effectiveness metrics	Key outcomes	Neurodevelopmental adaptations
Cognitive-behavioral therapy	Systematic reviews available (Tsubaki and Shimizu, 2024); Multiple RCTs	Mixed clinical populations; Limited ASD-specific studies	Moderate effect sizes (Cohen's *d* = 0.65) (Tsubaki and Shimizu, 2024); Pre-post improvements	Significant alexithymia reduction (Tsubaki and Shimizu, 2024); Mixed ASD results (Spain et al., 2015, 2017)	Requires autism-specific protocol modifications (Spain et al., 2015, 2017); Enhanced visual supports needed
Mindfulness-based interventions	RCT evidence (Ridderinkhof et al., 2018; Spek et al., 2013); Controlled trials	ASD populations, neurotypical samples	Large effect sizes (Cohen's *d* = 0.87) (Ridderinkhof et al., 2018); Sustained improvements	Enhanced emotional awareness (Ridderinkhof et al., 2018; Spek et al., 2013); Improved interoceptive processing (Gaigg et al., 2020; Cachia et al., 2016)	Demonstrates particular promise for ASD populations (Ridderinkhof et al., 2018; Spek et al., 2013); Sensory considerations are important
Emotion regulation training	Limited empirical studies (Conner et al., 2019; Mazefsky and White, 2014)	ASD adolescents and adults	Small-moderate effects (Cohen's *d* = 0.43) (Conner et al., 2019); Variable outcomes	Modest skill acquisition (Conner et al., 2019; Mazefsky and White, 2014); Individual variation in response	Requires individualized approach (Conner et al., 2019); Communication adaptations essential
Technology-assisted training	Emerging research (Dawson et al., 2010; Kasari et al., 2010); Preliminary trials	General alexithymic populations	Large effect sizes (Cohen's *d* = 0.97) (Lukas et al., 2019); Maintained follow-up gains	Significant emotion recognition improvements (Lukas et al., 2019); MT-ALEX app effectiveness (Fletcher-Watson et al., 2016)	High potential for neurodevelopmental adaptation (Lukas et al., 2021); Accessibility considerations needed
Social communication training	Limited research base (Cooper K. et al., 2018; Bemmouna et al., 2023)	ASD populations exclusively	Small effect sizes (Cohen's *d* = 0.38) (Cooper K. et al., 2018); Modest gains documented	Limited communication improvements (Cooper K. et al., 2018; Bemmouna et al., 2023); Context-specific benefits	Population-specific protocols required (Cooper K. et al., 2018); Integration with existing interventions needed

RCT, randomized controlled trial; Cohen's *d*, standardized mean difference effect size measure (0.2 = small, 0.5 = medium, 0.8 = large effect).

Cognitive-behavioral therapy (CBT) has emerged as the most extensively investigated approach, with systematic review evidence indicating moderate to large effect sizes (*d* = 0.65) across intervention studies (Tsubaki and Shimizu, 2024). However, only five studies examined CBT effectiveness in ASD populations with alexithymia, revealing mixed results and highlighting the need for autism-specific therapeutic adaptations (Spain et al., 2015, 2017). Traditional CBT protocols required substantial modification to address the unique cognitive and communication profiles of individuals with neurodevelopmental disorders.

Mindfulness-based interventions have demonstrated particular promise for improving emotional awareness and regulation in autism populations, with randomized controlled trials showing large effect sizes (*d* = 0.87) and clinically significant improvements in alexithymia scores following structured mindfulness training (Ridderinkhof et al., 2018; Spek et al., 2013). The mechanisms underlying mindfulness's effectiveness appeared to involve enhanced interoceptive awareness and improved attention to internal emotional states (Lukas et al., 2019, 2021).

Technology-assisted interventions represented an emerging and potentially transformative approach, with smartphone-based emotion recognition training showing exceptional effectiveness in preliminary trials (*d* = 0.97) (Lukas et al., 2019, 2021). The Mindtastic Alexithymia App (MT-ALEX) demonstrated superior outcomes compared with traditional therapeutic approaches, with sustained improvements at follow-up (Fletcher-Watson et al., 2016). However, no studies have examined technology-based interventions specifically in DS or dual-diagnosis populations, representing a significant development opportunity.

No intervention studies were identified that targeted alexithymia in Down syndrome or dual diagnosis populations, despite these groups potentially requiring specialized therapeutic approaches accounting for cognitive, communication, and behavioral characteristics unique to these conditions.

#### 3.3.4 Theme 4: neurobiological correlates and mechanistic understanding

Neurobiological research examining alexithymia in neurodevelopmental populations has revealed differences in brain structure and function, although the findings remain preliminary and have primarily focused on autism populations (Ernst et al., 2014; Donges and Suslow, 2017). Neuroimaging investigations have consistently implicated brain regions involved in emotional processing, including the anterior cingulate cortex, insula, amygdala, and prefrontal cortical areas (van der Velde et al., 2013; Karlsson et al., 2008).

Compelling mechanistic evidence emerged from functional magnetic resonance imaging (fMRI) studies, demonstrating that alexithymia, rather than autism *per se*, accounted for reduced activation in emotion-processing networks during facial emotion recognition tasks (Brewer et al., 2015; Bird et al., 2010). This finding fundamentally challenges traditional assumptions about emotional processing deficits in autism and supports the alexithymia hypothesis as an explanatory framework (Shah et al., 2016; Heaton et al., 2012), and may contribute to broader emotional processing difficulties in neurodevelopmental populations (Kano and Fukudo, 2013; Murphy et al., 2017).

The absence of neurobiological research in Down syndrome populations is concerning, with no studies identified examining brain correlates of alexithymia or emotional processing difficulties in this population.

#### 3.3.5 Theme 5: developmental trajectories and lifespan considerations

Previous studies have revealed substantial knowledge gaps, with most providing cross-sectional snapshots rather than longitudinal developmental perspectives (Frye et al., 2015; Billeci et al., 2016).

A 3-year longitudinal investigation by Oakley and colleagues followed 89 autistic adolescents, documenting moderate stability in alexithymia scores (*r* = 0.68) with considerable individual variation (Oakley et al., 2021). ~35% of participants showed meaningful improvement over time, while 28% demonstrated worsening alexithymia scores, and 37% remained stable. These findings suggest that alexithymic features are modifiable during development, supporting the potential for early intervention (Dawson et al., 2010; Kasari et al., 2010). This gap is particularly concerning given evidence that individuals with Down syndrome experience protracted emotional development and may not reach adult-level emotional regulation until their twenties or thirties (Wishart and Manning, 1996; Fidler et al., 2006).

#### 3.3.6 Theme 6: clinical implications and service delivery considerations

Clinical research examining the impact of alexithymia on service utilization, treatment outcomes, and quality of life in neurodevelopmental populations revealed implications for care delivery and support planning (Costa et al., 2019; Giannotti et al., 2020). Multiple studies in ASD populations demonstrated that alexithymic features significantly impacted therapeutic engagement and treatment outcomes across various intervention modalities (Gaigg et al., 2018; Mazefsky and White, 2014).

Individuals with co-occurring autism and alexithymia demonstrated reduced benefit from standard social skills interventions, emotion regulation training, and cognitive-behavioral therapy compared to autistic individuals without alexithymia (Spain et al., 2017; Gaigg et al., 2020). These findings suggest that addressing alexithymic features may be a prerequisite for effective intervention in many cases (Cooper K. et al., 2018; Bemmouna et al., 2023).

Quality-of-life research has consistently demonstrated negative associations between alexithymia severity and multiple life domains, including social relationships, educational achievement, vocational functioning, and independent living skills (Lai et al., 2013, 2015). However, quality-of-life research in DS populations remained extremely limited, with only one preliminary study examining relationships between emotional awareness and life satisfaction in this population.

An economic impact analysis revealed substantial costs associated with alexithymia in neurodevelopmental populations, including increased healthcare utilization, reduced educational outcomes, and reduced vocational productivity (Buescher et al., 2014; Rogge and Janssen, 2019). However, formal cost-effectiveness analyses of interventions for alexithymia were notably absent from the literature.

### 3.4 Research gap analysis

Seven critical research gaps emerged from this literature mapping, representing priorities for scientific advancement and clinical practice development:

Gap 1: Assessment Tool Validation Crisis—Current alexithymia measures demonstrate fundamental validity problems in intellectual disability populations (Greene et al., 2020; Kooiman et al., 2002; Levant et al., 2009), with no validated tools available for individuals with Down syndrome or severe autism spectrum disorder.

Gap 2: Down Syndrome Research Absence—Despite representing the most common genetic cause of intellectual disability (Sherman et al., 2007; De Graaf et al., 2015), DS populations remain virtually understudied regarding alexithymia prevalence, characteristics, and intervention needs.

Gap 3: Dual Diagnosis Population Invisibility—Complete absence of research examining alexithymia in DS-ASD populations, despite comprising 16-18% of DS individuals (DiGuiseppi et al., 2010; Capone et al., 2005) and potentially experiencing compounded difficulties.

Gap 4: Developmental Trajectory Vacuum—Longitudinal studies examining how alexithymic features emerge, develop, and change across the lifespan in neurodevelopmental populations are virtually non-existent (Frye et al., 2015; Billeci et al., 2016; Mazefsky et al., 2013).

Gap 5: Population-Specific Intervention Deficit—Limited intervention research specifically targeting alexithymia in neurodevelopmental disorders (Tsubaki and Shimizu, 2024; Gratz et al., 2014), focuses on general populations, with questionable applicability.

Gap 6: Cultural and Socioeconomic Homogeneity - The predominance of research conducted in high-income, English-speaking countries greatly limits generalizability across diverse cultural and socioeconomic contexts worldwide (Henrich et al., 2010; Nielsen et al., 2017).

Gap 7: Long-term Outcome Investigation Absence—Minimal research examines relationships between alexithymia and long-term adaptive functioning, quality of life, educational achievement, and vocational outcomes in neurodevelopmental populations (Howlin et al., 2004; Seltzer et al., 2004).

## 4 Discussion

### 4.1 Principal findings and scientific contributions

The synthesis demonstrates robust evidence for substantially elevated alexithymia prevalence in autism populations, with a weighted mean prevalence of 49.9% representing a 10.2-fold increase compared to general population rates (Kinnaird et al., 2019b). This finding is supported by multiple systematic reviews and large-scale investigations establishing the alexithymia hypothesis as a fundamental framework for understanding socio-emotional difficulties in autism spectrum disorder (Brewer et al., 2015; Bird et al., 2010; Shah et al., 2016).

However, this evidence of scientific progress contrasts with research imbalances that limit a comprehensive understanding and hinder the development of evidence-based practice. The disparity between autism and Down syndrome research represents an imbalance in neurodevelopmental science, given that Down syndrome affects ~250,000 individuals globally and constitutes the most common genetic cause of intellectual disability (Sherman et al., 2007; De Graaf et al., 2015). The complete absence of dual diagnosis research despite affecting 16-18% of individuals with Down syndrome (DiGuiseppi et al., 2010; Capone et al., 2005) highlights a population that remains scientifically invisible despite potentially experiencing complex emotional processing challenges requiring specialized assessment and intervention approaches.

A key theoretical question this review raises but cannot currently answer from existing data concerns the mechanistic pathways through which alexithymia may emerge differently in ASD vs. DS. In ASD, alexithymia is conceptualized as arising from atypical interoceptive processing and aberrant prediction error signaling in emotional networks, particularly the insula and anterior cingulate cortex (Brewer et al., 2015; Bird et al., 2010; Garfinkel et al., 2016). Reduced neural efficiency in representing internal affective states contributes to difficulty identifying feelings and to externally oriented thinking. In DS, the neurocognitive substrate differs substantially. Trisomy 21 is associated with a generalized reduction in processing efficiency and frontal-temporal hypoplasia rather than domain-specific representational differences (Lott and Dierssen, 2010; Menghini et al., 2010). This suggests that alexithymia in DS may emerge through a different route: reduced cognitive resources available for emotional introspection, rather than through atypical emotional representation *per se*. If confirmed, this mechanistic distinction would have direct implications for assessment and intervention design. Tools targeting interoceptive accuracy may be appropriate for ASD but less relevant for DS, where broader cognitive support strategies may be more effective. This distinction is currently theoretical and requires empirical investigation.

### 4.2 Implications for clinical practice and service delivery

The evidence mapping reveals implications for clinical practitioners working with neurodevelopmental populations, emphasizing the importance of screening for alexithymic features while acknowledging limitations in current assessment approaches (Mundy and Jarrold, 2010; Klin et al., 2015). The documented prevalence of ~50% in autism populations (Kinnaird et al., 2019b) necessitates routine alexithymia screening as standard clinical practice, with validated cutoff scores triggering specialized intervention protocols. However, the absence of validated assessment tools for individuals with intellectual disabilities creates practice challenges that require research attention (Tager-Flusberg and Kasari, 2013; Kasari et al., 2013).

For healthcare professionals working with autism populations, implement screening using the TAS-20 with appropriate caution regarding discriminant validity concerns (Preece et al., 2024; Leising et al., 2009), while recognizing that approximately half of autistic individuals experience clinically significant alexithymia levels, which should inform treatment planning (Volkmar et al., 2014; Bishop et al., 2015). Modified therapeutic approaches that emphasize emotion recognition training, development of interoceptive awareness, and enhancement of communication strategies have demonstrated effectiveness (Ridderinkhof et al., 2018; Spek et al., 2013).

An evidence-informed therapeutic pathway for neurodevelopmental populations with alexithymia would operate across three stages. In the first stage, targeted screening using the most appropriate validated tool for the population establishes baseline alexithymia severity: the TAS-20 for higher-functioning ASD; observer-rated scales for those with intellectual disabilities. In the second stage, intervention is matched to profile: mindfulness-based approaches for those with intact interoceptive awareness potential (*d* = 0.87) (Ridderinkhof et al., 2018; Spek et al., 2013); technology-assisted emotion recognition training for those with limited verbal engagement (*d* = 0.97) (Lukas et al., 2019); and modified CBT protocols for those with co-occurring mood dysregulation (*d* = 0.65) (Tsubaki and Shimizu, 2024). In the third stage, outcomes are monitored using standardized functional indicators, including quality of life, social participation, and therapeutic engagement, and the intervention is adjusted accordingly. For DS and DS-ASD populations, this pathway requires adaptation, given the complete absence of validated screening tools and intervention trials. Practitioners should currently implement modified observer-rated protocols and document outcomes to contribute to the emerging evidence base (Davis et al., 2018; Versaci et al., 2021).

Educational practitioners require guidance on identifying emotional processing difficulties in students with neurodevelopmental disorders, integrating brief screenings into existing health assessments, and developing individualized support plans that address emotion regulation and social communication challenges (Hume et al., 2021; Steinbrenner et al., 2020). For Down syndrome populations, practitioners must recognize the complete absence of validated assessment tools and implement preliminary screening using modified protocols and observer-based measures until validated tools become available (Davis et al., 2018; Versaci et al., 2021).

Clinical practitioners should also recognize that alexithymia likely presents differently across the ASD cognitive spectrum. Autistic individuals without intellectual disability may report alexithymia via self-identified difficulty labeling emotions and strong externally oriented thinking patterns, measurable with self-report tools such as the TAS-20 or PAQ (Bagby et al., 1994a; Preece et al., 2018). Those with co-occurring intellectual disability face an additional barrier: cognitive and language demands of self-report measures likely exceed their capacities, obscuring alexithymic features behind measurement artifacts (Levant et al., 2006; Sinason, 2005). Observer-rated behavioral indicators, including reduced spontaneous emotional language, limited affect-sharing, and restricted interoceptive responding, become the practical assessment targets in this group. This distinction is clinically critical: treating these presentations identically risks both underdetection in the lower-ability group and misattributing distress-driven responses to alexithymia in the higher-ability group.

### 4.3 Policy implications and system-level changes

The research imbalances and gaps identified through this synthesis necessitate policy responses that address funding priorities, research infrastructure development, and service delivery standards (Thornicroft et al., 2017; Kazdin and Blase, 2011). Healthcare policy should integrate alexithymia screening and intervention into quality indicators and accreditation standards, with specific requirements for neurodevelopmental disability services.

Funding agencies should prioritize research on Down syndrome and dual-diagnosis populations through targeted grant mechanisms that address the identified research gaps (Collins et al., 2011; Patel et al., 2018). Educational policy should include emotional processing assessment and intervention training in healthcare professional education programs, with competency requirements for working with neurodevelopmental populations (Shattuck et al., 2012; Roux et al., 2015). Healthcare systems should implement alexithymia-informed care models with specialized pathways for individuals with neurodevelopmental disorders, including modified therapeutic approaches, enhanced staff training, and outcome measurement systems sensitive to emotional processing challenges (Cawthorpe, 2018; Croen et al., 2006).

### 4.4 Scientific priorities and research advancement imperatives

The identified research gaps provide a roadmap for scientific advancement, with a priority focus on developing assessment tools, initiating research on Down syndrome, and investigating dual diagnosis populations. Priorities include developing and validating assessment measures suitable for individuals with intellectual disabilities, as well as using innovative approaches such as technology-assisted assessment, caregiver-report measures, and observational protocols (Aresti-Bartolome and Garcia-Zapirain, 2014).

Longitudinal developmental studies should be prioritized to understand how alexithymic features emerge, develop, and change across the lifespan in neurodevelopmental populations (Frye et al., 2015; Billeci et al., 2016). Large-scale, multi-site investigations with adequate statistical power are necessary to establish prevalence rates in Down syndrome and dual-diagnosis populations, while identifying both risk and protective factors (Buescher et al., 2014; Amaral et al., 2011).

Intervention research should focus on developing and testing population-specific therapeutic approaches (Tsubaki and Shimizu, 2024; Gratz et al., 2014), including modified cognitive-behavioral therapy protocols, technology-assisted interventions (Lukas et al., 2019, 2021), and family-centered approaches that address the unique needs of neurodevelopmental populations. Implementation research examining the barriers and facilitators to alexithymia screening and intervention in clinical settings highlights the need to translate research findings into practice (Brookman-Frazee et al., 2012; Stahmer et al., 2011).

### 4.5 Study limitations and analytical boundaries

This scoping review demonstrates limitations that must be considered when interpreting findings and developing research priorities. The search limitation to English-language publications potentially excluded relevant research from non-English speaking countries, particularly limiting representation from diverse cultural contexts (Kapp et al., 2013; Kenny et al., 2016). The heterogeneity in study designs, assessment tools, and population characteristics complicated synthesis and limited the ability to draw definitive conclusions about prevalence rates or intervention effectiveness (Pellicano et al., 2014; Pellicano and Stears, 2011).

The quality of included studies varied considerably, with many investigations suffering from small sample sizes, inadequate control groups, and insufficient statistical power to detect meaningful effects (Ioannidis, 2005; Button et al., 2013). The preponderance of cross-sectional studies limited understanding of developmental trajectories and long-term outcomes (Elman et al., 2013; Volkow et al., 2016).

However, the review was strengthened by search strategies that covered multiple databases and gray literature sources (Rethlefsen et al., 2021; Godin et al., 2015), selection procedures that followed established guidelines (Shea et al., 2017; Higgins et al., 2011), and data extraction processes with quality assessment (Liberati et al., 2009; Stroup et al., 2000). The broad scope enabled the identification of research gaps that might be overlooked in more focused reviews, providing valuable insights for research prioritization and funding allocation (Elman et al., 2013; Volkow et al., 2016).

## 5 Conclusion

Down syndrome populations remain understudied in alexithymia research, despite affecting ~250,000 individuals worldwide, representing a scientific and ethical priority requiring research attention and funding allocation. Research implications include the development and validation of assessment tools for individuals with intellectual disabilities, prevalence studies in Down syndrome populations, investigations into dual diagnoses, longitudinal developmental studies to understand lifespan trajectories, and methodological innovations such as technology-assisted assessment and cultural adaptations. Practice implications emphasize routine alexithymia screening in autism populations while recognizing assessment limitations in intellectual disability contexts. Evidence-based interventions demonstrating moderate to large effect sizes offer hope for improving outcomes, although population-specific adaptations require further development. Policy implications underscore the need for targeted funding to address research imbalances, healthcare policy that integrates alexithymia screening and intervention into quality standards, and educational policy that includes the development of emotional processing competency in professional training programs. Identifying seven research gaps provides a roadmap for scientific advancement. Addressing these gaps requires coordinated efforts across research institutions, funding agencies, clinical organizations, and policy bodies to ensure appropriate assessment, intervention, and support for emotional processing challenges across the neurodevelopmental spectrum.

## Data Availability

The raw data supporting the conclusions of this article will be made available by the authors, without undue reservation.
